# CocoaMoniliaDataSet: A cocoa pod dataset to detect and classify *Monilia roreri* in real conditions

**DOI:** 10.1016/j.dib.2025.112447

**Published:** 2026-01-07

**Authors:** Joan Alvarado, Juan Felipe Restrepo-Arias, David Velásquez, John W. Branch-Bedoya, Mikel Maiza

**Affiliations:** a Escuela de Ciencias Aplicadas e Ingeniería, Universidad EAFIT, 050022 Medellín, Colombia; b Facultad de Minas, Universidad Nacional de Colombia, 050041 Medellín, Colombia; c Fundación Vicomtech, Basque Research and Technology Alliance (BRTA), Mikeletegi 57, 20009 Donostia, San Sebastián, Spain

**Keywords:** Cocoa pod, Cocoa disease, Monilia disease, Image acquisition, Cocoa disease detection

## Abstract

Computer vision applications for detecting diseases in agriculture have been gaining relevance in recent years through the use of deep learning architectures. Digital image datasets serve as the main input for these architectures, enabling the analysis of patterns associated with a specific disease. However, some diseases have not yet been explored due to the limited availability of annotated image datasets. Cocoa pods are fundamental for the production of chocolate and its derived products; nevertheless, their production is threatened by *Monilia roreri*, a fungal disease responsible for yield losses of approximately 30% - 40%. Therefore, this paper proposes a CocoaMoniliaDataSet, a dataset of cocoa pods labeled across symptomatic stages of Monilia disease. Although the infection of cocoa pod caused by *Monilia roreri* describes four biological cycles, the dataset takes the visual symptoms into three classes to support computer vision task. These symptoms correspond to: cycle 1 (humps), cycle 2 (brown/oily spot and cycle 3 (white powder or sporulation). In this paper, cycle 2 represents a symptomatic stage that merges the second and third biological cycles. In addition, the dataset included healthy cocoa pods to facilitate early detection of the disease. The dataset comprises 1953 images with four labeled classes: (1) healthy cocoa pod labeled as (h0); (2) first Monlilia cycle, humps, labeled as (m1); (3) second - third Monilia cycle of the disease labeled as (m2); and (4) fourth Monilia cycle labeled as m3. Each instance in the image was annotated using the polygon method in CVAT (Computer Vision Annotation Tool), and the resulting labels are provided in COCO 1.0, YOLO, and segmentation mask 1.1 format to enable training object detection algorithms using bounding boxes. The publication of this dataset is essential for exploring techniques to diagnose cocoa disease using computer vision techniques.

Specifications Table

**Table:** 

Subject	Computer Sciences
Specific subject area	Detection of *Monilia Roreri* on cocoa pods.
Type of data	RGB Images, JSON files, YoLo annotations, and Mask annotations.
Data collection	The images were collected taking pictures with smartphone camera. Samsung Galaxy A24, Moto g54 5G, iPhone 14, Xiaomi-M1908C3JGG, Apple-iPhone 14, Xiaomi Redmi Note 9 Pro, and Lenovo Tab M10 Plus 3rd Gen.
Data source location	The data were collected at the "Compañía Nacional de Chocolates" farm, located in Santander, Colombia, and a local farm "Bosque Adentro" located in San Luis, Antioquia, Colombia.
Data accessibility	The dataset developed in this study is publicly available in the Zenodo repository [1] and it is distributed under Creative Commons Attribution 4.0 International License (CC BY 4.0). The dataset includes RGB images of cocoa pods affected by *Monilia roreri* and healthy cocoa pods, and the annotation files for each image. In addition, a README file is provided in the associated GitHub Repository, describing the structure of the dataset, directory organization, and example indicating how to load and use the CocoaMoniliaDataSet through a Google Colab notebook. The version of the dataset described in this manuscript corresponds to Zenodo DOI: 10.5281/zenodo.17716661. Repository name: Zenodo Data identification number: 10.5281/zenodo.17716661 Direct URL to data: https://zenodo.org/records/17716661 GitHub Repository: https://github.com/joanfco30/CocoaMoniliaDataSet
Related research article	None.

## 1 Value of the Data

Compared with existing datasets, this work introduces the following novel aspects:

• The dataset contains images of cocoa pods at different stages of *Monilia roreri* disease, each one with its corresponding label. This multi-class representation allows the development of computer vision models to computer vision models to identify transitions between cocoa disease cycles.
• Unlike existing publicly datasets on cocoa disease, such as Cocoa Diseases (YOLOv4), which is scarce and generally limited to binary classifications (healthy vs unhealthy), the CocoaMoniliaDataSet provides an annotation scheme with four classes, providing more detailed representation of the Monilia disease progression. This multi-class structure supports the early detection and enables the development of deep-learning models capable of differentiating intermediate symptomatic stages.
• The dataset will be publicly available and can be used by both public and private institutions to support the diagnosis of diseases in cocoa crops and to explore new monitoring strategies, such as mobile diagnostic applications for farmers and automated monitoring system based on computer vision.
• The dataset serves as input for state-of-the-art computer vision algorithms aimed at providing automated solutions in cocoa crop diagnosis and supporting research on object detection, instance segmentation and classification tasks. The availability of annotations in multiple formats (COCO 1.0, YOLO, and segmentation masks) improves accessibility and enables immediate integration into various deep learning pipelines without requiring additional preprocessing or format conversion steps.
• The labeled images enable the automatic detection of cocoa pod conditions in real-world applications, improving the performance of detection and classification tasks and supporting digital agriculture tools to detect Monilia disease in cocoa.

## 2 Background

The detection and classification of cocoa diseases has become a field of interest in agriculture because it provides tools to prevent production losses. Cocoa production is the foundation of the chocolate industry, but it is threatened by several diseases that affect its production chains [2]. One of these diseases is the *Monilia roreri*, a fungal pathogen that directly infects cocoa pods. The disease is common in the Central and Latin America region, which is responsible for causing economic and crop losses of 30% - 80% of their total annual production [3]. In recent years, the use of deep learning architectures in computer vision has evidenced strong potential for disease detection in plants. However, even though there are studies that explore computer vision to detect disease in cocoa crops, there is still under exploration due to the scarcity of annotated datasets.

In computer vision applications, cocoa diseases such as Monilia and Phytophthora are among the most commonly reported in the literature [4]. Several classification tasks using computer vision have been studied to detect and classify cocoa diseases. For example, Aamoako et al. [5] proposed a classification approach based on the VGG-19 Convolutional Neural Networks to classify the cocoa pods into three categories (Phytophthora, Monilia, and healthy). Likewise, Soh et al. [6] evaluated the performance of different deep learning architectures for the classification of Black Pod Rot in cocoa pods. Beyond the use of deep learning architectures, traditional machine learning methods such as Support Vector Machine or Random Forest have been explored to differentiate between healthy and diseased cocoa pods [7,8]. Nevertheless, most of the datasets used in computer vision to classify cocoa pods are only classified as healthy or diseased [9], without capturing the progressive cycle of Monilia, which is essential for early detection of the disease. The CocoaMoniliaDataSet was created to address this limitation, providing several clone varieties of cocoa pods such as CNCH12, CNCH13, SCI-1, and TRINITARIO [10,11], labeled across three cycles of the Monilia, as well as healthy pods. The datasets support the training of deep learning algorithms based on computer vision to detect diseases in real agricultural conditions. are the most common diseases reported in literature.

## 3 Data Description

**Fig. 1:**
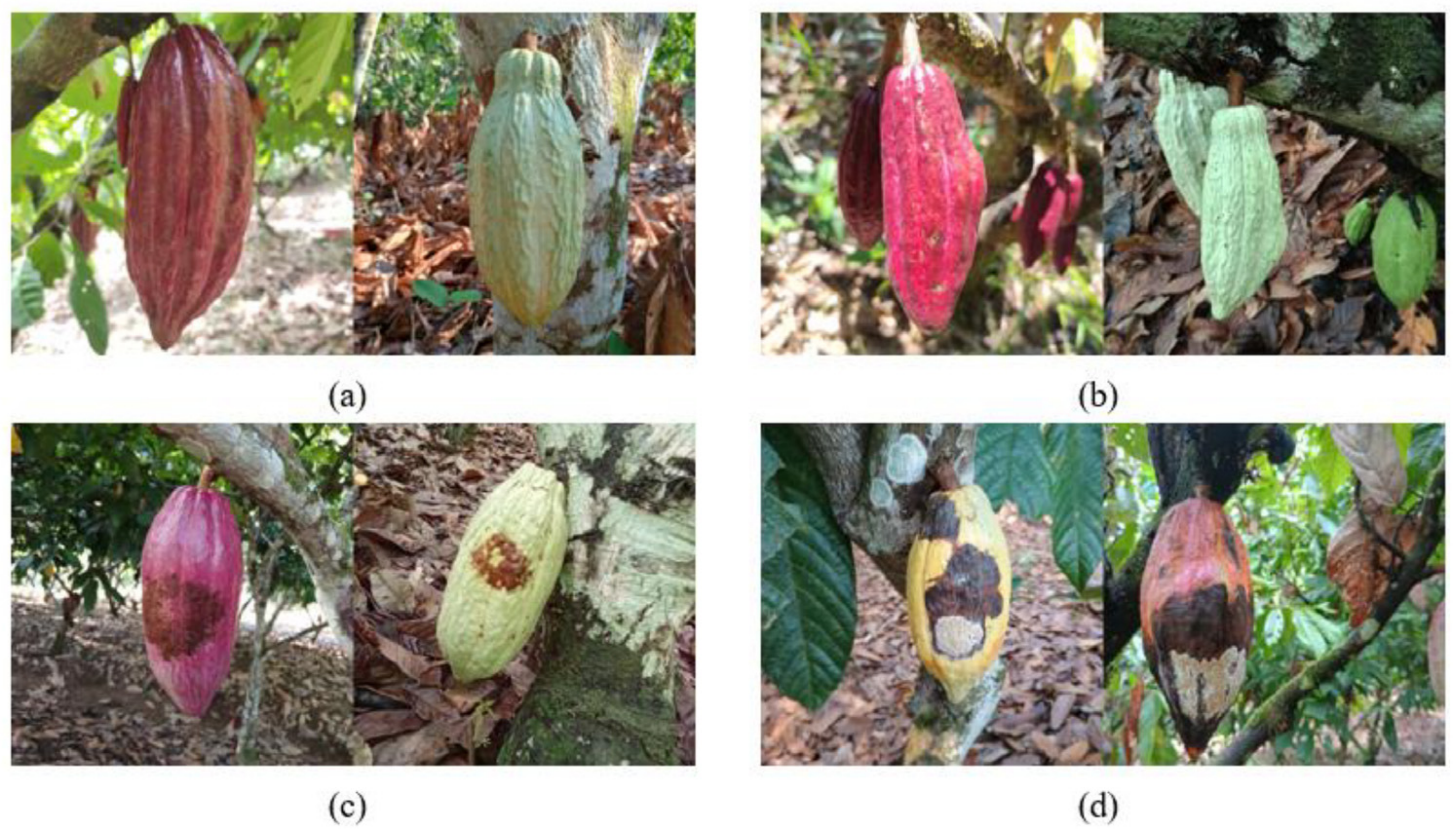
Cocoa pods examples of the four classes included in the CocoaMoniliaDataSet.: (a) Healthy pods (h0); (b) Cocoa pods in the first cycle with visible humps (m1); (c) Cocoa pods in the second–third cycle showing brown/oily spot (m2); (d) Cocoa pods in the fourth cycle characterized by white powdery sporulation (m3).

The dataset was created with a total of 1953 images in JPG format and 2123 annotated instances, where each instance corresponds to an individual cocoa pod within the images. According to the technical information provided by the Compañía National Chocolate [9,11], this dataset involved the labeling of four classes: (1) Healthy cocoa pods labeled as h0, (2) First cycle of Monilia (Humps) labeled as m1, (3) corresponds to the second - third cycle of Monilia (brown/oily spot) labeled as m2, and (4) Fourth cycle of Monilia (Sporulation) labeled as m3. It should be noted that, class m2 merges the second and third cycle of the Monilia disease. These classes were selected because they reflect the main symptomatic stages of Moniliophthora roreri disease, allowing for optimized early detection applying computer vision techniques. Overall, the total size of the dataset is approximately 6.2 GB. Fig. 1 shows an illustrative example of the cocoa pods dataset, categorized in four classes. Fig. 1a illustrates healthy cocoa pods, labeled as h0. Fig. 1b shows pods affected by the Monilia disease, characterized by humps. Fig. 1c displays the cocoa pods exhibiting the brown/oily spot stage. Finally, Fig. 1d depicts the cocoa pods with advanced disease presenting sporulation. These images illustrate the visual variability within each class, including differences in size, shape and symptom severity.

**Fig. 2:**
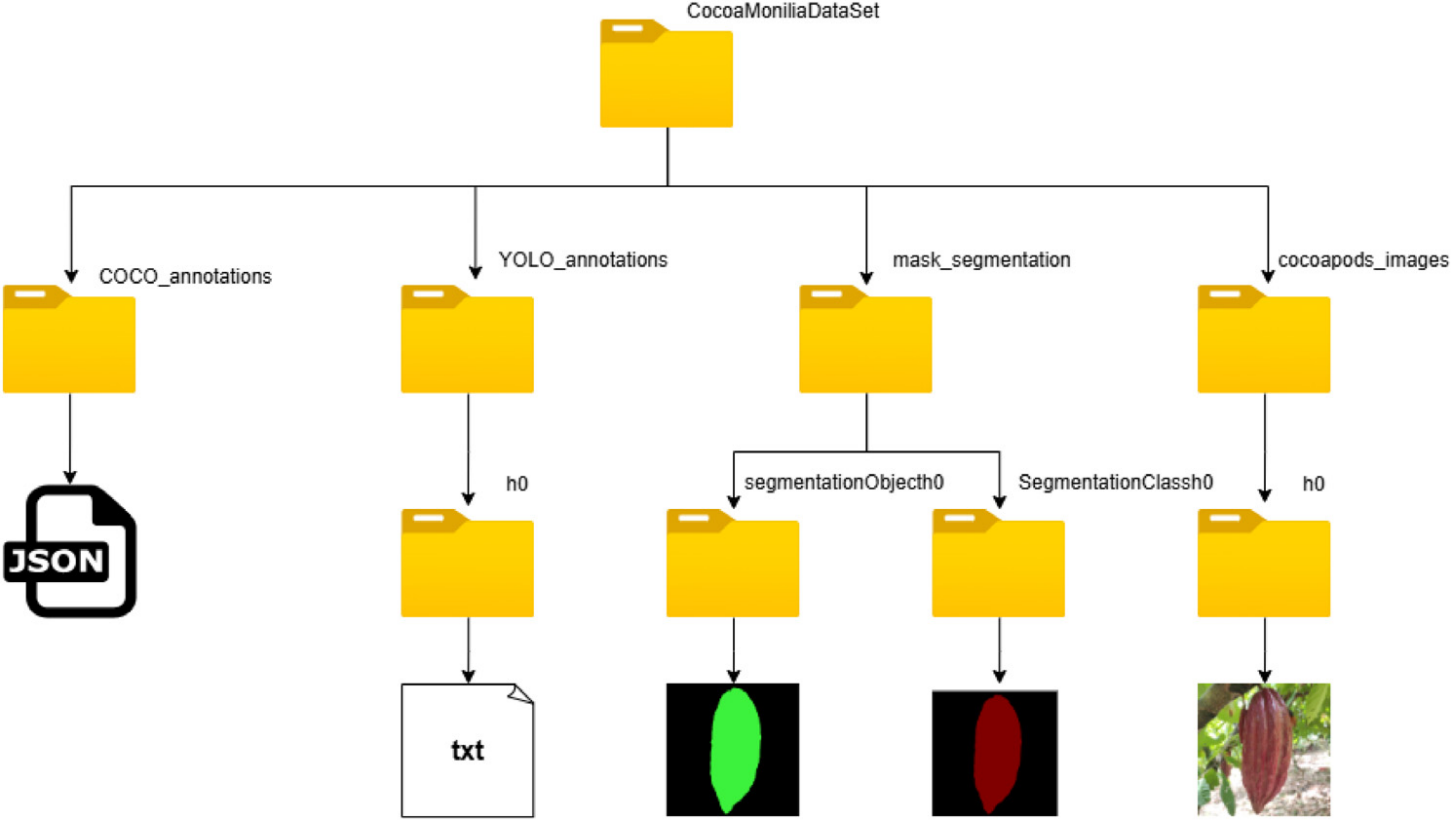
Structure of the directory CocoaMoniliaDataSet, including the organization of images, COCO and YOLO annotations formats, and segmentation mask.

Fig. 2 provides an overview of how the CocoaMoniliaDataSet is structured. The organization of dataset directories. depicted in Fig. 2, is arranged as follows: In the parent directory, four directories were created. The first, called COCO_annotations, this directory contains the annotations of the cocoa pods represented in four JSON files called *instances_h0.json, instances_m1.json, instances_m2.json, and instances_m3.json*. The next directory was called YOLO_annotations, inside the YOLO_annotations directory there are four directories named h0, m1, m2, and m3 with the YOLO annotations inside them, this annotation consist in a (.txt) which contain the class and the location of the cocoa pods according to its respective image.

Then mask_segmentation directory includes the annotations in segmentation mask 1.1 format, into the directory, there are two main sub-groups, SegmentationClass and SegmentationObject, each one is divided into four directories according to the class of the dataset. The image files of these directories contain the semantic and instance segmentation annotations in (.png) format. Finally in Fig. 2, the directory cocoapod_images is split into four directories: h0, m1, m2, and m3, which store the original images in (.jpg) format linked to the respective annotation files. CocoaMoniliaDataSet includes predefined training (80%), validation (10%), and testing (10%) splits, provided in (*.txt) files into the repository to ensure reproducible and comparable evaluations.

**Fig. 3:**
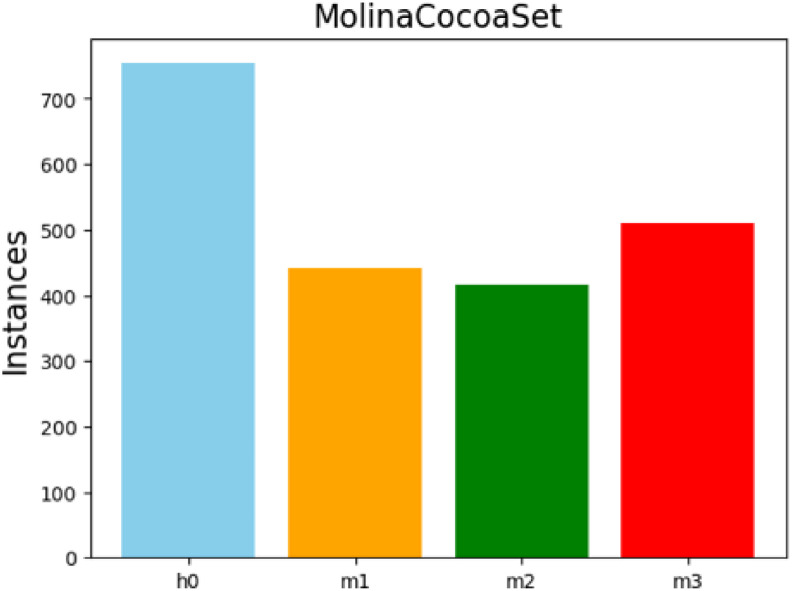
Distribution of classes in the dataset across the four classes of the CocoaMoniliaDataSet.

Table 1 summarizes the number of annotated instances within the dataset, which comprises a total of 1953 images. The largest class corresponds to healthy cocoa pods (h0) with a total of 754 instances. The state of the cocoa pods with humps (m1) accounts for 442 instances, while those affected by brown/oily spot (m2) comprise 416 instances. Finally, the cocoa pods affected by sporulation are represented by 511 instances. Fig. 3 illustrates the distribution of annotated instances across the four classes of the CocoaMoniliaDataSet, providing a graphical representation of the counts reported in Table 1.

**Table 1:** Distribution of annotation instances (N = 2123) across the four classes.

Classes	h0	m1	m2	m3
Instances	754	442	416	511

Image acquisition process was carried out under different variable conditions, lighting conditions (morning and afternoon), different angles, pod orientations, and background complexity caused by branches and leaves.

Finally, Table 2 summarizes the technical details (metadata) of the CocoaMoniliaDataSet, including the image characteristics, image resolution, image format, cocoa pod varieties and other features of the dataset.

**Table 2:** Summary of CocoaMoniliaDataSet metadata.

Feature	Description
Total images	1953
Total annotated instances	2123
Image format	RGB (JPG)
Annotation format	COCO 1.0, YOLO, Segmentation Mask 1.1
Annotation tool	CVAT (Computer Vision Annotation Tool)
Annotation method	Polygon
Cocoa varieties	CNCH12, CNCH13, SCI-1, and TRINITARIO
Locations	Santander & Antioquia, Colombia
Classes	h0 (healthy), m1 (cycle 1 – humps), m2 (cycle 2–3 – brown/oily spot) and m3 (cycle 4 – sporulation)
Dataset size	∼6.2 GB

## 4 Experimental Design, Materials and Methods

The images collected for the dataset were taken in two farms or cocoa plantations located in Colombia during the period between September/2024 and February/2025. The first plantation, "Granja Yariguíes - Compañía Nacional de Chocolates", is located in vereda La Lejia, Barrancabermeja, department of Santander. In this farm, images collected at this site included CNCH12, CNCH13, SCI-1, and TRINITARIO cocoa pod clone varieties [10,11]. The second location, "Bosqueadentro", is a local farm located in Vereda San Pablo, San Luis, department of Antioquia, where the images included TRINITARIO varieties [10]. The images were taken using RGB cameras during the morning between 8:00 and 12:00, and afternoon between 14:00 and 16:00 sessions to include the sunlight and weather conditions, except for rainy conditions. In addition, these images were captured from different angles, and they contain stems, branches, leaves, and cocoa pods.

Images were captured using different smartphone cameras such as Samsung Galaxy A24, Moto g54 5G, iPhone 14, Xiaomi-M1908C3JGG, Apple-iPhone 14, Xiaomi Redmi Note 9 Pro, and Lenovo Tab M10 Plus 3rd Gen. All pictures were taken at the default resolution of each device from 8MP to 12MP, producing images with dimensions of 3024*3024px, 3072*3072px, 3474*3472px, and 2992*4000px in JPG format. The camera functions were configured in automatic mode (AUTO) to adjust the exposure (aperture, shutter speed, and ISO sensitivity) of the picture in real light conditions. In addition, flash mode was disabled to keep the natural light environment.

**Fig. 4:**
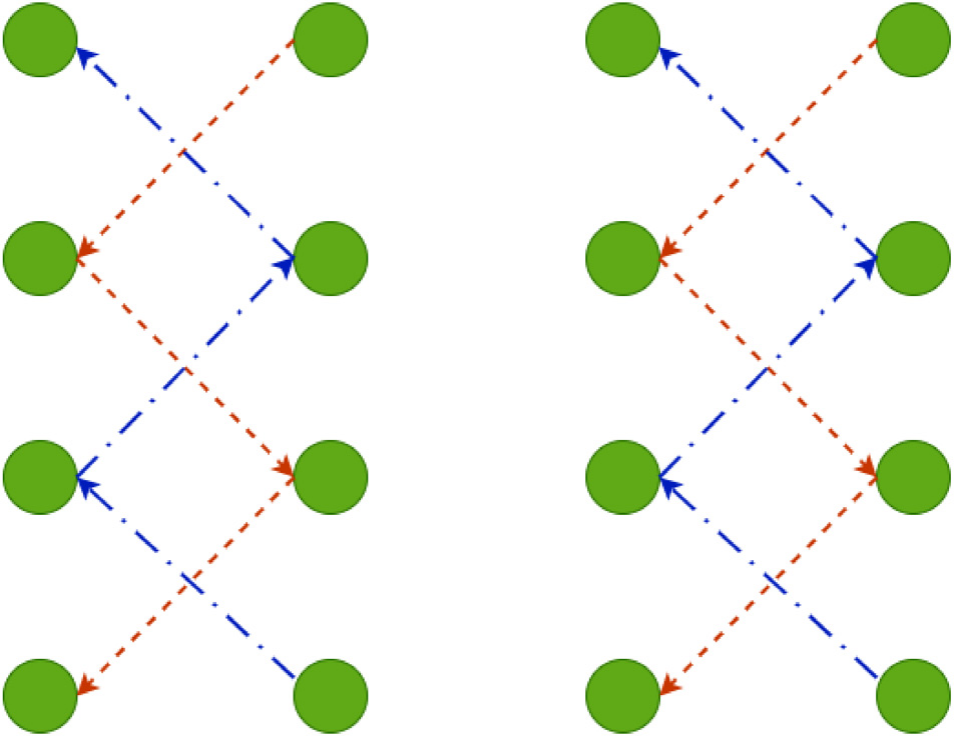
Scheme proposed for capturing images at each cocoa tree within a plantation.

To ensure that images of each cocoa tree are captured in an organized manner, a systematic route through the crop is proposed, following a zigzag pattern along the field. This procedure consists of walking through the crop following predefined paths that ensure complete coverage of all trees without omissions or unnecessary repetitions. Fig. 4 shows the systematic method designed to take pictures at each cocoa tree within a plantation. In the image, the green circles denote the cocoa trees. According to this method, two walking methods were proposed: The orange trajectory follows a zigzag pattern across consecutive trees, while the blue trajectory alternates diagonally between rows. At designated points, individual cocoa trees were selected, and photographs of the pods were captured.

**Fig. 5:**
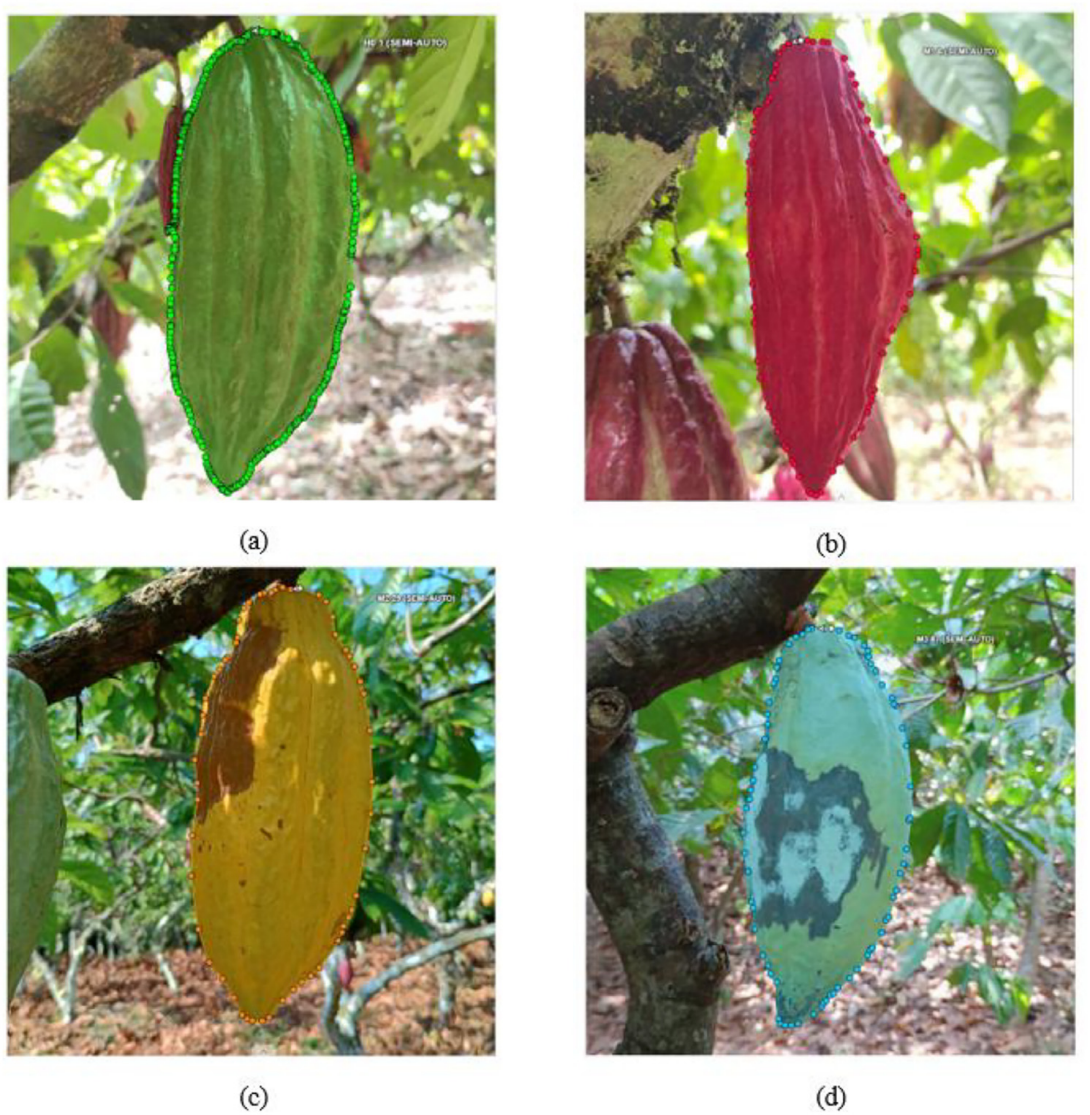
Examples of cocoa labeling with Computer Vision Annotation Tool (CVAT): (a) Polygon labeling healthy pods;(b) Polygon labeling cocoa pod affected by humps; (c) Polygon labeling cocoa pod affected by brown/oily spot; (d) Polygon labeling cocoa pod affected by sporulation.

The tool employed for labeling images was CVAT (Computer Vision Annotation Tool) [12]. The annotation process was conducted manually, and each image was labeled according to its predefined class (h0, m1, m2, and m3) using the polygon labeling technique to obtain pixel-level semantic segmentation of the classes. This annotation process consists of delineating the exact contour of the cocoa pods by sequentially positioning points along their edges and connecting them to form a closed shape. Fig. 5 shows an example of the annotation technique employing polygon labeling. Figs. 5a, 5b, 5c, and 5d show the result obtained after applying polygon annotation on each image, where the outline of the pod was manually delimited. In addition, this process was carried out in collaborations with experts from the Compañía Nacional de Chocolates following the guideline “LA MONILIASIS DEL CACAO: DAÑOS, SÍNTOMAS, EPIDEMIOLOGÍA Y MANEJO” [13] to ensure consistency in the annotations.

**Fig. 6:**
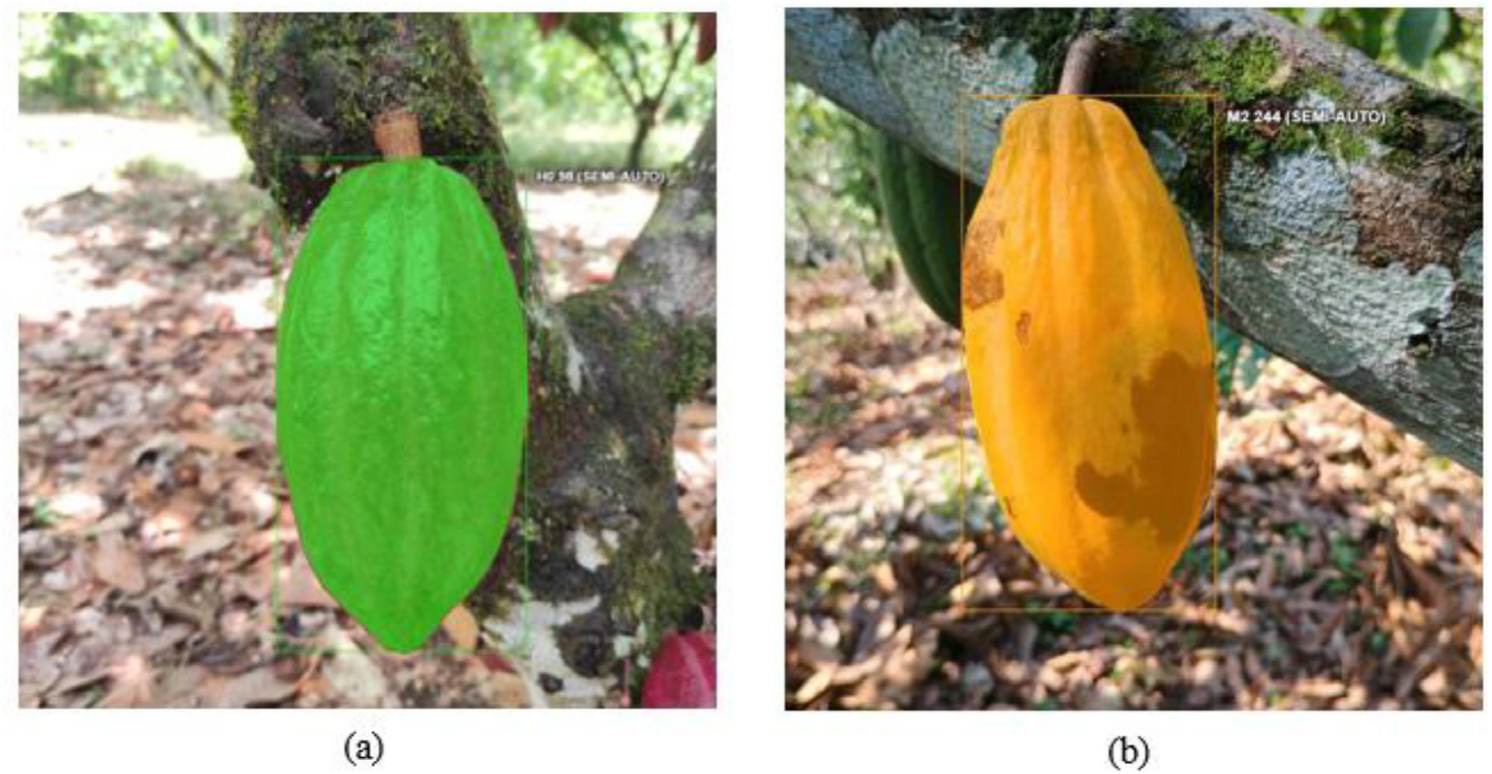
Examples of cocoa labeling with Computer Vision Annotation Tool (CVAT): (a) Bounding box labeling healthy pods; (b) Bounding Box labeling cocoa pod affected by brown/oily spot.

The dataset follows the annotation scheme in COCO 1.0, YOLO, and segmentation mask 1.1 format. The first scheme has files in the format (*.json) which include information on bounding box coordinates, polygon segmentation, categories, and image IDs. The second has files in (*.txt) format, which include annotations in YOLO format, where each file corresponds to an image. Fig. 6 depicts an example of a cocoa bounding box read by the (*.txt) files. As observed in Fig. 6, the top right of the box is referenced with its respective class. Fig. 6a shows an example of a bounding box annotation labeled as ho (healthy), and Fig. 6b shows the bounding box annotation labeled as m2 (brown/oily spot).

Segmentation mask 1.1 annotation format includes separate directories of images in PNG format for semantic segmentation and instance segmentation.

**Fig. 7:**
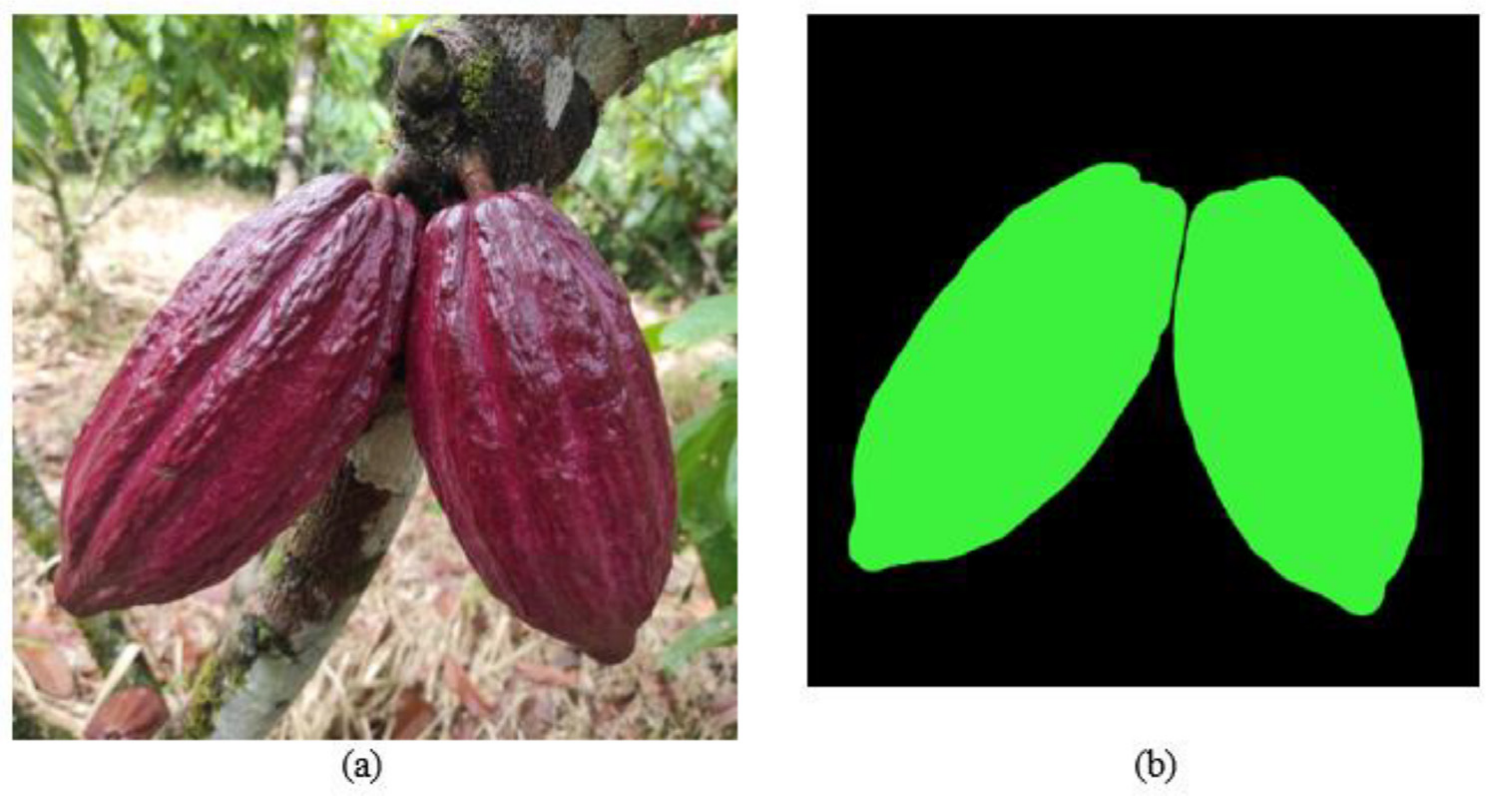
Examples of masks for semantic segmentation with Computer Vision Annotation Tool (CVAT): (a) Original RGB image in (.jpg) format of healthy pod; (b) Image of mask for semantic segmentation of healthy pod.

**Fig. 8:**
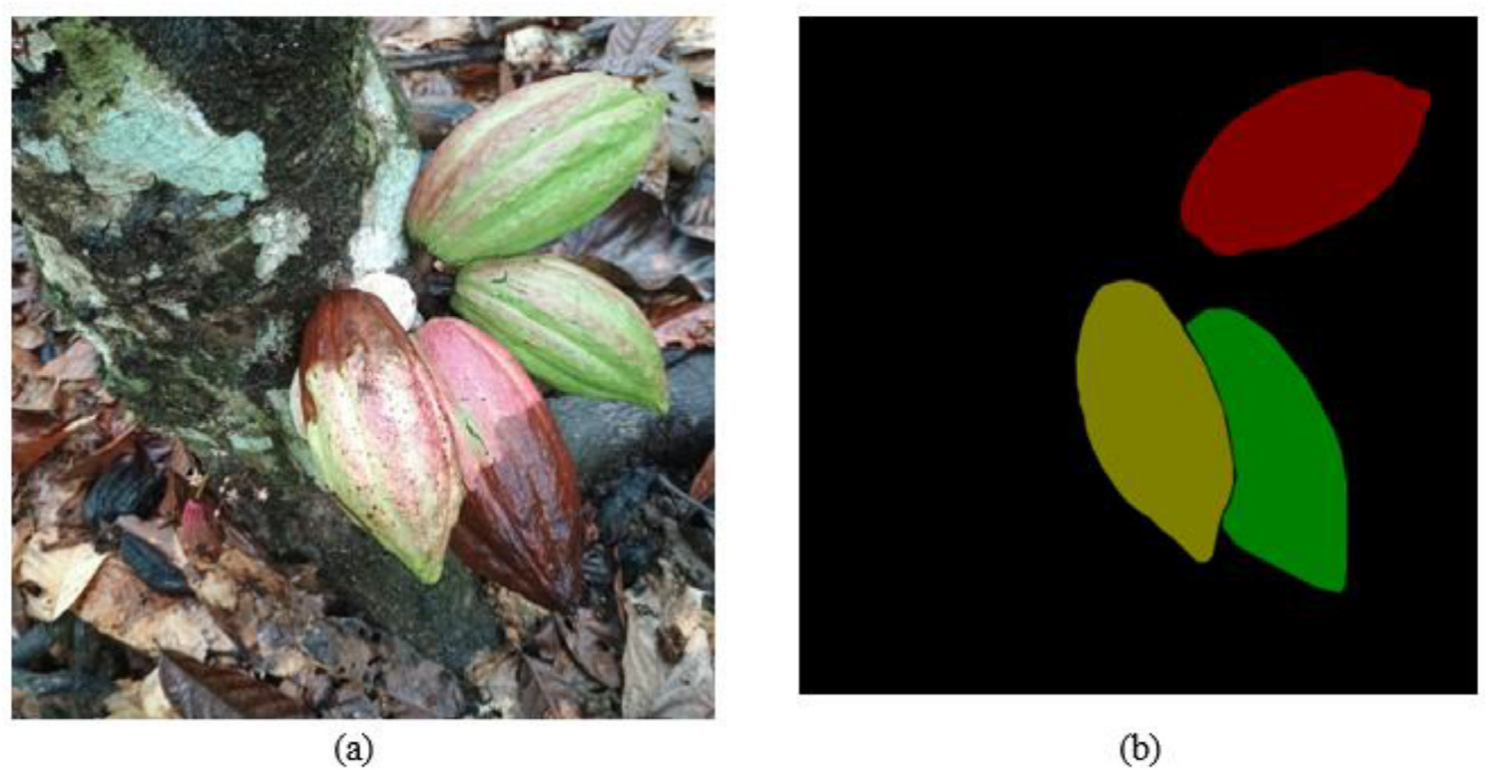
Examples of masks for instance segmentation with Computer Vision Annotation Tool (CVAT): (a) Original RGB image in (.jpg) format of healthy and disease cocoa pod; (b) Image of mask for instance segmentation of healthy and disease cocoa pod.

Fig. 7, Fig. 8 show examples of mask segmentation generated with CVAT. Fig. 7a shows the original RGB image in format (.jpg) of a healthy cocoa pod, while Fig. 7b presents the mask of the image for semantic segmentation, which is stored in (.png) format. In addition, Fig. 8a depicts the original image used for instance segmentation, and Fig. 8b displays its corresponding instance segmentation masks.

Finally, Table 3 presents the label generated for the segmentation mask 1.1. As evidenced in Table 3, the first column represents the classes, each of which is represented by a unique RGB color value to facilitate the pixel differentiation.

**Table 3:** Label map of the segmentation mask 1.1 annotation.

Classes	RGB color value
h0	(61,242,60)
m1	(250,50,83)
m2	(245,147,49)
m3	(50,183,250)

The CocoaMoniliaDataSet does not include information about personal data, human subjects or sensitive data. All images exclusively depict cocoa pod and cocoa plantations.

## Limitations

The CocoaMolinaDataSet is only focused on the Monilia diseases in cocoa pods. In future work, diseases such as phytophora may be included and explored.

## Ethics Statement

the authors have read and follow the ethical requirements for publication in Data in Brief and confirming that the current work does not involve human subjects, animal experiments, or any data collected from social media platforms.

## Credit Author Statement

**Joan Alvarado**: Conceptualization, Methodology, image capture, labeling, data curation, writing. **Juan Felipe Restrepo-Arias**: Supervision, original draft preparation, image capture, labeling, reviewing. **David Velásquez**: Supervision, original draft preparation, reviewing. **John W. Branch-Bedoya**: Supervision. **Mikel Maiza**: Supervision.

## Data Availability

ZenodoCocoaMoniliaDataSet (Original data).
